# Cerebral photoreception in mantis shrimp

**DOI:** 10.1038/s41598-018-28004-w

**Published:** 2018-06-26

**Authors:** Mary W. Donohue, Jonathan H. Cohen, Thomas W. Cronin

**Affiliations:** 1 Department of Biological Sciences, University of Maryland Baltimore County, Baltimore, Maryland 21250 USA; 2 School of Marine Science and Policy, College of Earth, Ocean and Environment, University of Delaware, Lewes, Delaware, 19958 USA

## Abstract

The currently unsurpassed diversity of photoreceptors found in the eyes of stomatopods, or mantis shrimps, is achieved through a variety of opsin-based visual pigments and optical filters. However, the presence of extraocular photoreceptors in these crustaceans is undescribed. Opsins have been found in extraocular tissues across animal taxa, but their functions are often unknown. Here, we show that the mantis shrimp *Neogonodactylus oerstedii* has functional cerebral photoreceptors, which expands the suite of mechanisms by which mantis shrimp sense light. Illumination of extraocular photoreceptors elicits behaviors akin to common arthropod escape responses, which persist in blinded individuals. The anterior central nervous system, which is illuminated when a mantis shrimp’s cephalothorax protrudes from its burrow to search for predators, prey, or mates, appears to be photosensitive and to feature two types of opsin-based, potentially histaminergic photoreceptors. A pigmented ventral eye that may be capable of color discrimination extends from the cerebral ganglion, or brain, against the transparent outer carapace, and exhibits a rapid electrical response when illuminated. Additionally, opsins and histamine are expressed in several locations of the eyestalks and cerebral ganglion, where any photoresponses could contribute to shelter-seeking behaviors and other functions.

## Introduction

Photoreceptors provide animals with information that can be used for vision, photoentrainment, reflex responses, or other functions. Nearly all known animal photoreceptors utilize visual pigments, made of transmembrane opsin proteins bound to a light-sensitive chromophore, to detect light. Outside of eyes, opsins are often associated with dermal or nervous tissues^1^. These opsin-based extraocular, “nonvisual” photoreceptors have been implicated in functions such as photoentrainment, escape responses, orientation, and reproduction^2–7^.

Among the taxonomically diverse arthropods, some species (*e.g*. dragonflies, horseshoe crabs, and mantis shrimps) express more than a dozen opsins, and some species (*e.g*. horseshoe crabs and crayfishes) are known to possess extraocular photoreceptors^4,5,8–11^. Within Pancrustacea, the two cephalic photoreceptive organs—image-forming lateral eyes and simpler median eyes—and expressed opsins have a particularly rich evolutionary history^12^. Median eyes, also called nauplius or ventral eyes, are common among Malacostraca, a class of crustaceans within Pancrustacea that includes crayfishes, crabs, mantis shrimps, etc. Transparent pelagic malacostracan crustacean larvae have a rudimentary ventral eye associated with the cerebral ganglion (CG), or brain, that may be used for simple visual tasks, like orientation (recently reviewed by Cronin *et al*.^6^). The ventral eye persists in the adults of some crustacean species, including gonodactyloid, pseudosquilloid, and squilloid mantis shrimps^13^. Mantis shrimp ventral eyes are anatomically reduced compared to the ventral eyes described in other malacostracan crustaceans^13^, and their functionality as extraocular photoreceptors has not been tested.

Mantis shrimps typically inhabit shallow coral reefs, where their vision is critical for intraspecific communication, prey capture, and other interactions. Opsin-based visual pigments and optical filters produce up to 16 physiologically-distinct photoreceptor classes used in UV, color, polarization, spatial, and motion vision^14–20^. The large number of opsins expressed in the eyes, and the resulting diversity of photoreceptors, is thought to reflect rampant gene duplications^18^. We recently found four opsins that are expressed in the CG of adult mantis shrimp (*Neogonodactylus oerstedii*)^21^. However, it was unknown whether or not functional photoreceptors exist in the CG or elsewhere in the central nervous system.

We looked for opsin-based cerebral photoreceptors in *N. oerstedii* to investigate the possible functions of extraocular, “nonvisual” photoreceptors. Here, we report that mantis shrimp react when illuminated by tail-flipping, walking, or swimming. These behaviors persist in animals that have been blinded. We also detected an electrical response from a putative ventral eye. We found expression of two opsins in the ventral eye, and two opsins elsewhere in the CG and optic lobes. The opsins have distinct expression patterns, which implies that they are involved in numerous functions. Light detection is an important sensory modality in mantis shrimp, and we show that it extends beyond their eyes.

## Results

### Evidence for functional extraocular photoreceptors

**Figure 1:**
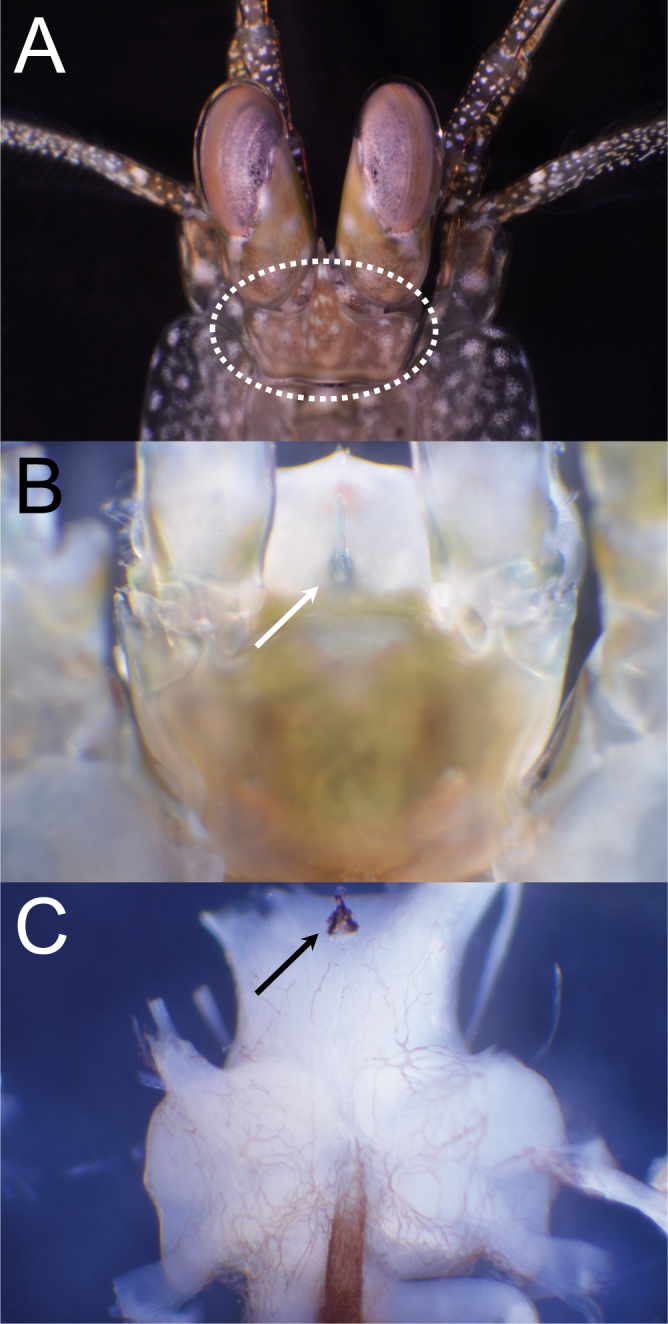
The pigmented ventral eye and the cerebral ganglion (CG), or brain, of *Neogonodactylus oerstedii*. (**A**) The CG is housed within the cephalothorax behind the eyes (oval). (**B**) A ventral view of an illuminated cephalothorax reveals a slightly darker region (arrow) that corresponds to the ventral eye location on the surface of the isolated CG. (**C**) A prominent pigmented structure (arrow) is visible toward the anterior end on the ventral surface of an isolated CG of adult *N. oerstedii*. The ventral eye we identified in an adult mantis shrimp is similar in location^6^ to the ventral eye in mantis shrimp larvae.

**Figure 2:**
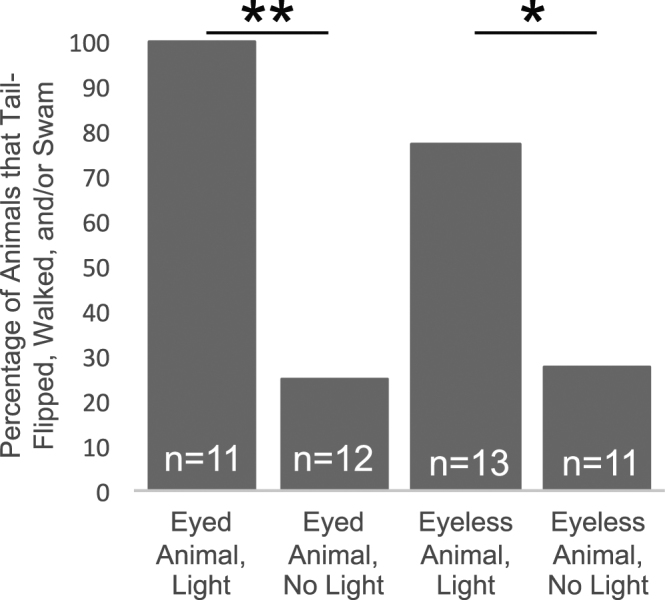
Behavioral evidence for extraocular photoreception in mantis shrimp. Whole-body illumination elicits common escape responses (tail-flipping, walking, and swimming) in normal and blinded mantis shrimp. These behaviors were observed less frequently when mantis shrimp are left in the dark without burrows. *P < 0.05; **P < 0.01.

We predicted that mantis shrimp have functional extraocular photoreceptors in the cephalothorax (Fig. 1). First, we quantified behavioral responses of eyed and eyeless *N. oerstedii* mantis shrimp to identify any potentially extraocular photoreceptor-mediated behaviors. We found that both eyed and eyeless animals tail-flipped, walked, or swam (see Methods section for description) when illuminated following dark-adaptation. Representative videos showing the responses of eyed and eyeless individuals to illumination are available as Supplementary Videos S1 and S2, respectively. All eyed mantis shrimp showed one or more of these responses, whereas 76.9% of eyeless mantis shrimp responded according to our criteria (Fig. 2). These values were significantly greater than the percentage of either eyed or eyeless animals that exhibited any of these behaviors when maintained in the dark during control trials of equal duration (25.0% and 27.3% for eyed and eyeless animals, respectively). However, the time it took before an animal responded following the onset of illumination, termed the response latency, was significantly longer for eyeless individuals ($\bar{x}$ of 6.7 s and 26.5 s and σ of 4.28 and 19.31 for eyed and eyeless individuals, respectively; Mann-Whitney U test *P* < 0.05).

**Figure 3:**
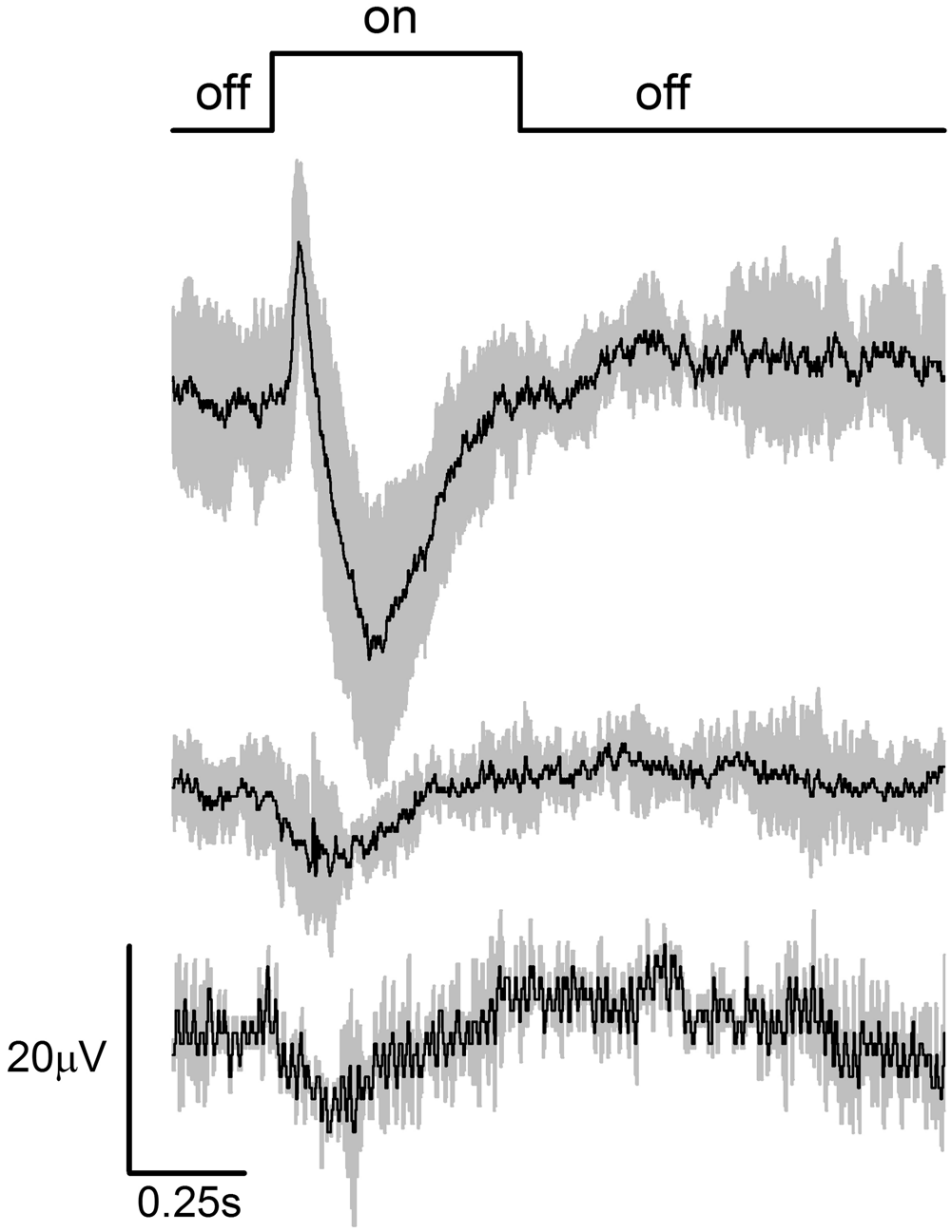
Electrophysiological evidence for extraocular photoreception in mantis shrimp. Illumination of the eyeless mantis shrimp cephalothorax produced electrical responses in AC recordings from the ventral eye. Shown are data for three preparations that varied in response magnitude upon stimulation with the same 0.5 s white light flash (6.14 × 10^15^ photons cm^2^ s^1^). The onset of light stimulation is indicated by the “on” phase of the positive square wave. Black lines are mean ERG response (n = 2−5 light flashes per preparation), grey shading is standard deviation.

To test for cerebral photosensitivity, we recorded extracellularly from the pigmented putative ventral eye of eyeless adult mantis shrimp. The putative cerebral photoreceptors exhibit an electrical response within 31 ms (±12 ms, sd) of bright white illumination, reaching peak magnitude in 169 ms (±59 ms, sd) (Fig. 3). A corneal positive (downward) ERG was present in all specimen tested, with an additional pronounced corneal negative (upward) ERG noted in one specimen. The latter response occurs in recordings from the crustacean retina, and could result here from differences in electrode depth among preparation and consequently either second-order neuron responses or contributions from photoreceptor cells with multiple spectral classes (*e.g*.^22^,). Sensitivity of the preparations varied and was too low overall to determine spectral sensitivity or to fully characterize the temporal dynamics of cerebral photoreceptors.

### Mantis shrimp extraocular photoreceptor molecular machinery

Following our observations of light-induced behaviors in eyeless mantis shrimp, and the identification of a photosensitive ventral eye, we investigated the expression patterns of opsins potentially used to produce light sensitive visual pigments in mantis shrimp extraocular photoreceptors. Our previous transcriptomic analyses revealed that four opsin transcripts—one middle-wavelength-sensitive (MWS) and three long-wavelength-sensitive (LWS) opsins—are expressed in the CG of *N. oerstedii*^21^. Therefore, we labeled mantis shrimp eyestalk and CG tissues using gene-specific *in situ* probes to visualize the expression of these four identified opsin transcripts.

**Figure 4:**
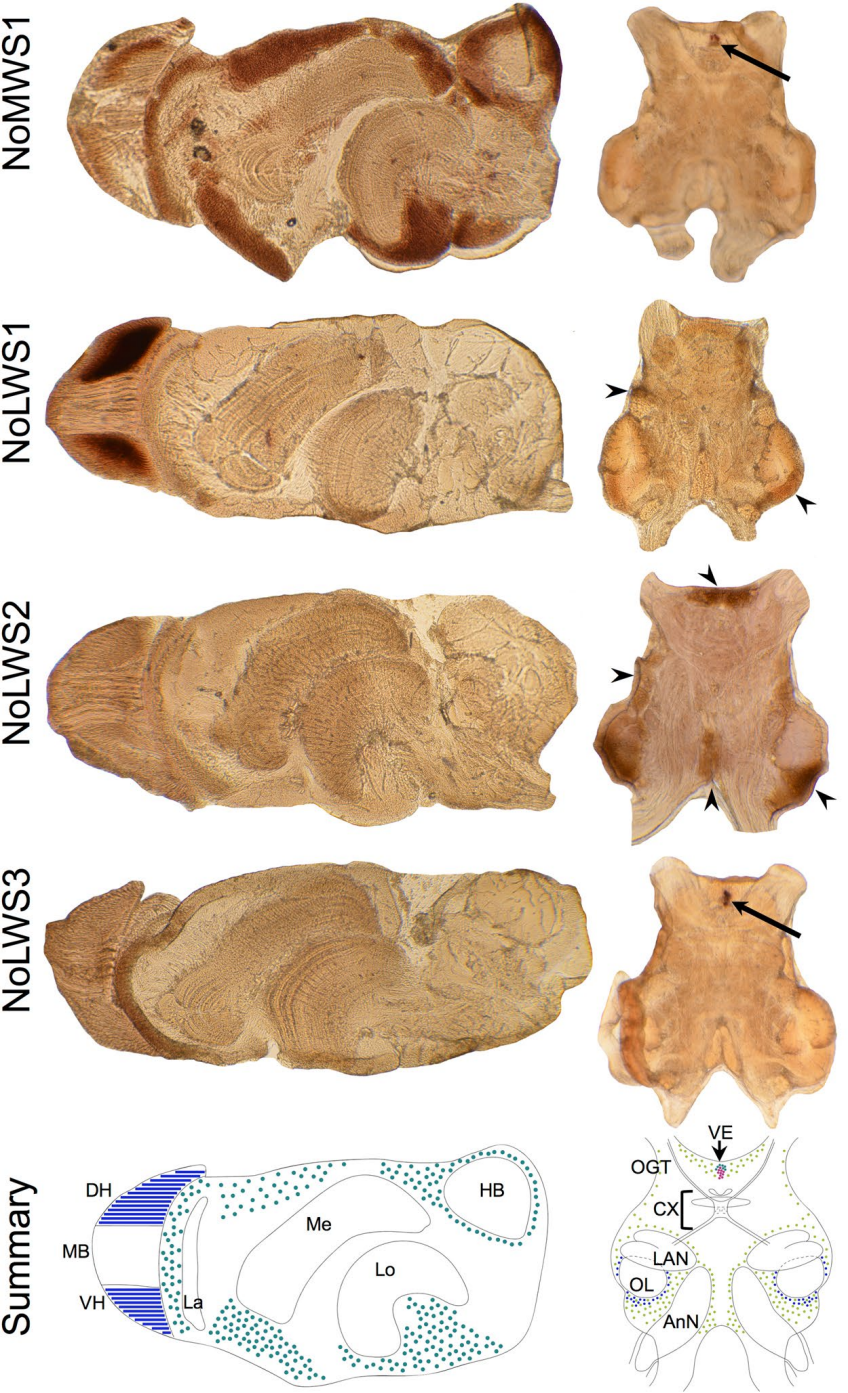
Opsin transcripts are expressed in the anterior portion of the central nervous system of *Neogonodactylus oerstedii*. *In situ* hybridization labeling patterns of 60 μm sagittal optic lobe sections (left column) and transverse cerebral ganglion (CG) sections (right column) show that of the four opsin transcripts expressed in the CG, only two are expressed within the retina and/or neural tissue within the eyestalk carapace. NoMWS1 is expressed in cell bodies surrounding several neuropils—the lamina (La), medulla (M), lobula (Lo), and hemiellipsoid body (HB)—within the eyestalk, and may be expressed in the most dorsal photoreceptors in the retina. NoLWS1 riboprobe labels the dorsal hemisphere (DH) and ventral hemisphere (VH) of the retina, but not the equatorial midband (MB) rows. Within the CG, NoMWS1 and NoLWS3 are expressed in the ventral eye (VE, arrows), which lies anterior to the central complex (CX). NoLWS1 and NoLWS2 are expressed in several regions with densely-packed, large cell bodies (arrowheads) that surround the antennal neuropil (AnN), olfactory lobe (OL), lateral antennular neuropil (LAN), and olfactory-glomeruli tract (OGT). An anatomical diagram summarizes NoMWS1 (teal), NoLWS1 (dark blue), NoLWS2 (green), and NoLWS3 (purple) transcript expression in the optic lobes and CG.

In the retina and eyestalk, the riboprobes for NoMWS1 and NoLWS1, but not NoLWS2 and NoLWS3, label tissues (Fig. 4). The NoMWS1 opsin riboprobe labels the outer portion of retinal photoreceptors in the dorsal hemisphere and cell bodies surrounding several neuropils (the lamina, medulla, lobula, and hemiellipsoid body) within the eyestalk. The NoLWS1 opsin riboprobe labels the dorsal and ventral hemispheres of the retina, likely labeling transcripts of No14, an opsin transcript sequence that was isolated from the eye of *N. oerstedii*^18^ and has the same 3′ untranslated region (3′-UTR) sequence (see ref.^21^ for sequence alignment). In the CG, NoMWS1 and NoLWS3 opsin transcripts are coexpressed in the ventral eye, and NoLWS1 and NoLWS2 opsin transcripts are expressed in cell bodies, including those in the olfactory lobe (Fig. 4). No labeling was observed in sense probe or no probe controls (Supplementary Fig. S1).

**Figure 5:**
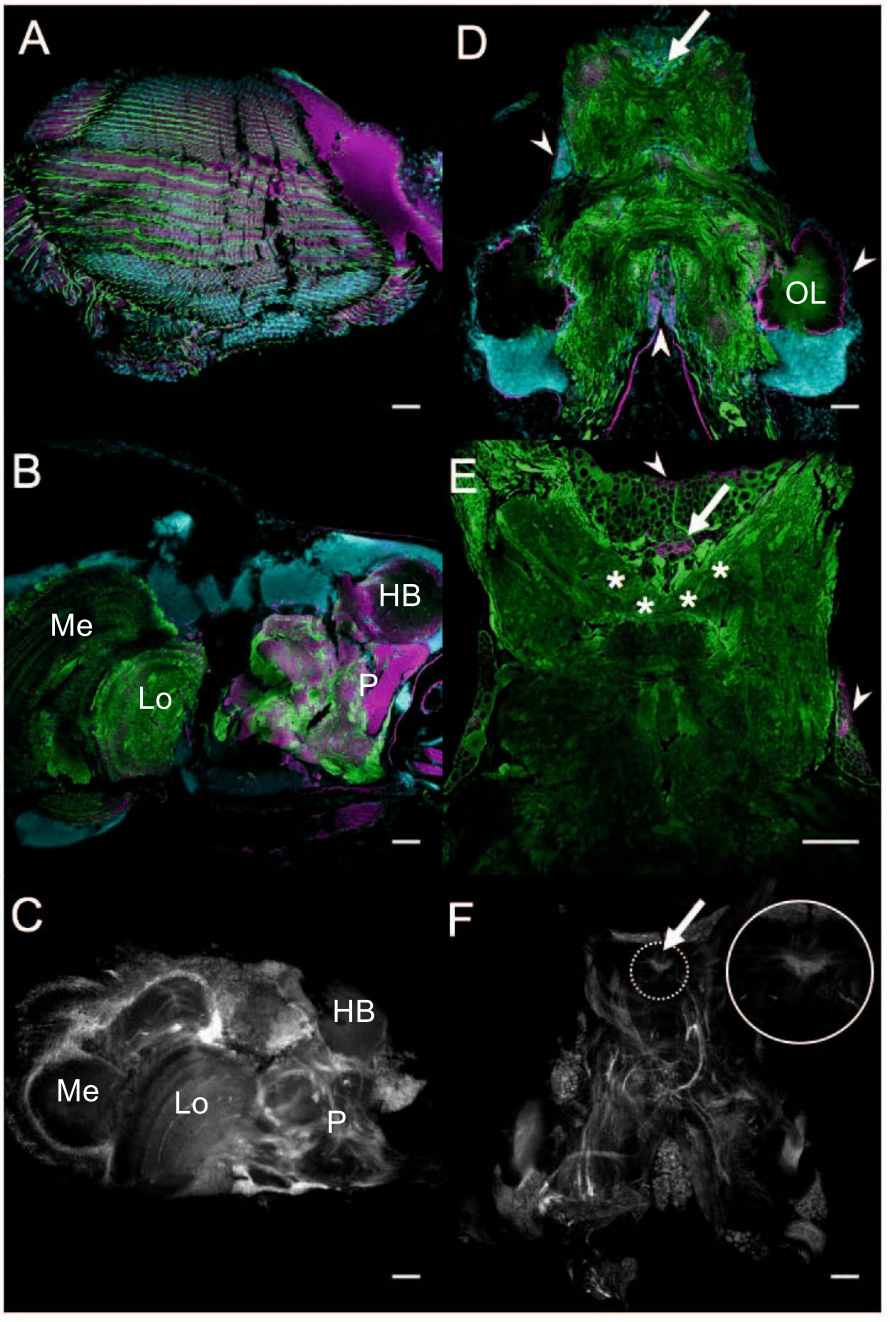
Labeling patterns of antibodies designed to characterize opsin protein (magenta) and histamine (white) expression are consistent with *in situ* labeling of opsin transcripts. Cytoskeletal tubulin and cell nuclei are visualized using anti α-tubulin antibody (green) and DAPI (cyan), respectively. (**A**) A coronal retinal section shows that antibody designed against crayfish long-wavelength-sensitive opsin labels all photoreceptors in the mantis shrimp (*Neogonodactylus oerstedii*) retina. (**B**) A sagittal view of the optic lobe shows that anti-opsin antibody labels the hemiellipsoid body (HB) and protocerebrum (P). (**C**) A sagittal section of the optic lobe shows that anti-histamine antibody labels tissue throughout the optic lobe, including the protocerebrum. (**D**) A transverse section of the cerebral ganglion (CG) reveals anti-opsin antibody labeling of the ventral eye (arrow), dense clusters of cell bodies (arrowheads), and periphery of the olfactory lobes (OL). (**E**) The ventral eye (arrow) is visualized in a transverse CG section and appears to extend large neuronal bundles to posterior brain regions (asterisks). The anti-opsin antibody also labels cell bodies around the periphery of the CG (arrowheads). (**F**) Anti-histamine antibody labeling is consistent with anti-opsin antibody labeling throughout the transverse CG section, including the VE (arrow, inset in F). Lobula (Lo); medulla (M).

To investigate whether or not the four opsin transcripts expressed in the anterior portion of the central nervous system produce opsin proteins, we utilized an antibody raised against a crayfish LWS opsin, which binds to proteins in stomatopod eyes and cerebral tissues with approximately the same molecular weight (50 kDa) as invertebrate opsins^23^ (Supplementary Fig. S2). The anti-opsin antibody labels the same structures in the mantis shrimp retina, eyestalks, ventral eye, olfactory lobes, and cerebral regions with clusters of large cell bodies (Fig. 5A,B,D,E) that express opsin transcripts (Fig. 4). An anti-histamine antibody, which labels the nauplius eye in a copepod^24^, also labels the same neural regions as the anti-opsin antibody in mantis shrimp (Fig. 5C,F).

## Discussion

Our discovery of at least one functional cerebral photoreceptor expands the understanding of mechanisms by which mantis shrimp sense light. Our data imply that, in addition to eyes specialized for detecting a variety of visual cues, mantis shrimp also possess extraocular “nonvisual” photoreceptors that can elicit escape responses common to aquatic arthropods. The light-induced behaviors we observed in eyeless mantis shrimp are similar to caudal photoreceptor-mediated behaviors in crayfish^11^. Common among decapods, caudal photoreceptors are thought to utilize opsins^23^, but were not found in the only mantis shrimp species examined to date (*Squilla empusa*)^5^. Mantis shrimps typically inhabit burrows that shield the body from light. However, when peering out of the shelter, the mantis shrimp’s cephalothorax (and any cerebral photoreceptors within) is illuminated by light. The mantis shrimp ventral eye may be homologous to other crustacean medial eyes, but could be functionally analogous to decapod caudal photoreceptors. Still, the presence of extraocular photoreceptors beyond the cerebral ones we found warrants further investigation, as currently undescribed non-cerebral photoreceptors may elicit the behavioral responses we described.

Opsins that are diffusely expressed in mantis shrimp optic lobes probably function separately from the structured medial photoreceptors of the ventral eye. Opsins expressed in arthropod optic lobes are thought to entrain biological rhythms because they are coexpressed with clock genes in bumblebees^25^, and are coexpressed with arrestin, chaoptin, and melatonin in hawkmoths^26^. Extraocular photoreceptors in mantis shrimp could affect circadian photoentrainment or biological rhythms (*e.g*. visual rhythms described by Cronin^27^) via the neurosecretory sinus gland complex called the x-organ, which is situated within the optic lobes of the eyestalk. Because mantis shrimp are typically shaded by their burrows throughout the day any photoreceptors used to properly entrain biological rhythms would probably be located within the illuminated cephalothorax extending from the burrow. Taken together, the expressed MWS opsin in mantis shrimp optic lobes is probably involved in circadian rhythm photoentrainment to some degree.

Diffusely expressed opsins in the mantis shrimp CG could be involved in circadian photoentrainment, multisensory integration, or other functions. In crayfish, two clusters of opsin-based photoreceptors are associated with the CG and have been implicated in circadian photoentrainment^28^. The cerebral photoreceptors in crayfish extend projections to the protocerebral bridge^28^, a neuropil of the central complex that provides input to the circadian rhythm-regulating sinus gland^29^. Since then, the arthropod central complex has been compared to the vertebrate basal ganglia, affecting behavior in the form of motor output, memory formation, attention, and sleep^30^. As we recently suggested^21^, information from opsin-based extraocular photoreceptors could be integrated with other sensory input in the central complex (or elsewhere) to affect various aspects of an animal’s physiology. Observed opsin expression patterns in mantis shrimp are consistent with this hypothesis. Opsins are expressed in neuropils of the optic lobes and CG that receive input from sensory appendages, and in a ventral eye that is proximal to the central complex. However, direct evidence of opsins expressed in extraocular tissues functioning in this capacity awaits investigation. Both MWS and LWS opsin transcripts are expressed in extraocular tissues, so any associated visual pigments are expected to be maximally sensitive to light with wavelengths of 400 nm or longer. More focused physiological or behavioral experiments could test this hypothesis.

The roles of opsins expressed outside of eyes has been debated, but the particularly dynamic history of pancrustacean opsins^12^ may reflect a variety of specialized locations and functions. Such seems to be the case in mantis shrimp; the segregation of extraocular opsins and any associated photoreceptors in specific neural structures are indicative of specialized functions. The light-avoidance behaviors we observed likely reveal only one of several roles in which mantis shrimp extraocular photoreceptors are involved. Beyond the ventral eye that may be used for shelter-seeking behaviors, opsins are abundant in neuropils in the eyestalk and CG, where they may be involved in circadian photoentrainment, multisensory integration, or other physiological roles. Future research on the ecology and evolution of extraocular photoreception in stomatopods is an intriguing prospect.

## Materials and Methods

### Behavior

Mantis shrimp (*Neogonodactylus oerstedii*) from the Florida Keys, Florida, USA were kept in plastic aquaria with approximately 800 ml artificial seawater (Instant Ocean Sea Salt) and were maintained in a 12-hour light: 12-hour dark cycle. At least 24 hours prior to experimentation, a subgroup of mantis shrimp were blinded by removing the eyes at the base of the eyestalk with ShearCut scissors. Immediately prior to a dark-adaptation period and subsequent experimentation, the burrow was removed from the individual’s aquarium, and the aquarium was arranged so that it could be illuminated by an infrared light (and white light for experimental trials). Throughout all dark-adaptation periods and trials, an infrared light illuminated the animal’s aquarium, so that behaviors could be monitored even in the dark. Mantis shrimp were dark-adapted for 1 h (9–10 AM) and then exposed to white light from a flood lamp, or kept in the dark for control trials, for two minutes. Trials were recorded using either a camcorder or an infrared-sensitive camera coupled to a recorder. The videos were then reviewed for the presence or absence of three behaviors: tail-flipping, walking, and swimming. A behavior was classified as tail-flipping if the animal was observed flexing at the thoracomeres, lifting its telson over its body, and somersaulting to face in the direction opposite its starting position; a behavior was classified as walking if there was a change in the animal’s location on the surface of the aquarium floor; and a behavior was classified as swimming if all pereiopods lifted off of the aquarium floor. Significance between treatments was determined by χ^2^ analyses. The response latency—the time between the onset of light and the exhibition of tail-flipping, walking or swimming—was recorded for trials during which animals responded to a light stimulus.

### Electrophysiology

To record extracellular electrical activity solely from the mantis shrimp ventral eye, the compound eyes were removed at the base of the eyestalk using ShearCut scissors. Animals were then mounted onto wooden craft sticks with cyanoacrylate gel adhesive applied along the dorsal carapace. The ventral eye was then exposed by removing the ventral carapace of the cephalothorax, and the animal held in a static, room-temperature seawater bath. Recording generally followed Charpentier and Cohen^31^. Electrical responses were recorded using a metal microelectrode (125 μm shank; FHC, Bowdoinham, ME, USA) placed beneath the screening pigment and microvilli of the ventral eye. A differential electrode was placed in the seawater bath. The differential AC signals were amplified (EXT-02 B, NPI Electronic, Tamm, Germany), digitized, and stored (PowerLab 8/35, LabChart software, AD Instruments, Colorado Springs, CO, USA). Light from a 175 W xenon arc lamp (Spectral Products, Putnam, CT, USA) was directed using a fiber optic light guide to illuminate the cephalothorax, and a computer-driven electromagnetic shutter provided a 500 ms flash of white light (6.14 × 10^15^ photons cm^−2^ s^−1^) with stimulus irradiance further controlled by fixed neutral density filters (Melles Griot, Rochester, NY, USA).

### Tissue preparation

Mantis shrimp (*N. oerstedii*) from the Florida Keys, Florida, USA were immediately sedated upon arrival to the University of Maryland, Baltimore County using cold artificial seawater. The animals were decapitated by a transverse cut across the cephalothorax posterior to the cerebral ganglion (CG) and anterior to the subesophageal ganglion. The eyestalks, antennules, antennae, and antennal scales were removed, and the CG and surrounding tissues were then fixed overnight at 4 °C in 4% paraformaldehyde (PFA) and 12% sucrose in 0.1% diethyl pyrocarbonate (DEPC) 1x phosphate-buffered saline (PBS). Tissues were then transferred to 0.1% DEPC 1x PBS for at least 24 h before the CG was dissected out of the partially encapsulating carapace. The CG was dehydrated (using an ethanol gradient, followed by propylene oxide) and rehydrated (using an ethanol gradient) before embedding the tissue in gelatin using a plastic mold. Blocks of gelatin were fixed overnight in 4% PFA in 0.1% DEPC 1x PBS and then transferred to 0.1% DEPC 1x PBS. Gelatin-embedded tissue was sectioned with a vibratome at 60 μm for *in situ* hybridization or immunohistochemical experiments described below.

### *In situ* hybridization

Custom genes designed against the 3′-UTR of the four opsins identified by Donohue *et al*.^21^, flanked by restriction enzyme and polymerase sites, were transformed into DH5α *Escherichia coli* bacteria. The sequences used as templates to label NoMWS1, NoLWS1, NoLWS2, and NoLWS3 are provided in Supplementary Table S1. The plasmids from successfully transformed bacteria were purified, and sequences confirmed by sequencing. Digoxigenin (DIG)-labeled probes were then created from the plasmid DNA using NotI restriction enzyme and T3 RNA polymerase for antisense labeling probes, and EcoRI restriction enzyme and T7 RNA polymerase for sense control probes.

The 60 μm sections (as described in the Tissue preparation section) were placed in glass wells and fixed in 4% PFA in 0.1% DEPC 1x PBS for 10 minutes, washed in 0.1% DEPC 1x PBS for 3 min (3x), and 0.1% DEPC H_2_O for 3 min (1x). Sections were then acetylated for 10 min in a solution of DEPC H_2_O, triethanolamine, 6 N hydrochloric acid, and acetic anhydrate. Tissue was then washed in 0.1% DEPC 1x PBS for 5 min (3x). Tissue was incubated in hybridization solution of 50% formamide, 5x saline-sodium citrate buffer (SSC), 5x Denhardt’s solution, 250 μg/ml tRNA, and 500 μg/ml herring sperm DNA at room temperature for 1 h before applying hybridization solution containing 0.25 to 1 ng probe per μl hybridization solution and incubating at 71 to 72 °C overnight. Tissue was then incubated in 0.2x SSC for 20 min (3x). Sections were equilibrated in a Tris and sodium chloride buffer, blocked with normal goat serum (NGS) at room temperature, and incubated in 1:5000 anti-DIG antibody for 48 h at 4 °C. After rinsing in the Tris and sodium chloride buffer, the sections were equilibrated in a Tris, sodium chloride, and magnesium chloride buffer. Samples were then incubated in a nitro-blue tetrazolium/5-bromo-4-chloro-3′-indolyphosphate (NBT/BCIP) and levamisole solution for 48 h at 4 °C before being transferred to DEPC H_2_O until they were photographed under a light microscope. Gelatin surrounding the tissue (which looked the same in all sections) were removed from the images using Photoshop.

### Immunohistochemistry

Gelatin-embedded sections were placed on a shaker for all incubations. Sections were washed in 0.5% Triton in 1x phosphate-buffered saline (PBS-TX) for 20 min (5x), blocked in PBS-TX with 10% NGS for 1 h, and incubated in primary antibody (1:1000 dilutions for anti-α-tubulin and anti-opsin antibodies, and 1:500 dilution for histamine) diluted in PBS-TX with 10% NGS at room temperature overnight. The sections were again washed in PBS-TX for 20 min (5x) before being incubated overnight at room temperature in secondary antibody (1:400) diluted in PBS-TX with 10% NGS. Sections were rinsed in PBS for 20 min (5x) before being mounted on slides with DAPI Fluoromount (Southern Biotech, Birmingham, AL, USA). Sections were then imaged using an SP5 Leica Confocal Microscope and processed in ImageJ. Western blots confirmed that the anti-opsin antibody binds protein approximately 50kDA in size (Supplementary Fig. S2), the size of similar crustacean opsins^23^. Secondary-only controls were performed in the same manner, but were incubated in PBS-TX with NGS without primary antibody (Supplementary Fig. S3).

### Data availability

All data are available upon request.

## Electronic supplementary material

Supplementary Figures

Video - Supplementary Video S1

Video - Supplementary Video S2
