# Supplementary material for: Cerebral photoreception in mantis shrimp
Source: Sci Rep. 2018 Jun 26;8:9689. doi: 10.1038/s41598-018-28004-w (PMC6018774; doi:10.1038/s41598-018-28004-w)
Supplement: Supplementary file 1 — Supplementary Figures [file 41598_2018_28004_MOESM1_ESM.docx]

**Title:** Cerebral photoreception in mantis shrimp

**Authors:**

Mary W. Donohue^1^

Jonathan H. Cohen^2^

Thomas W. Cronin^1^

^1^Department of Biological Sciences, University of Maryland Baltimore County, Baltimore, Maryland 21250, USA

^2^School of Marine Science and Policy, College of Earth, Ocean and Environment, University of Delaware, Lewes, Delaware 19958, USA

**Correspondence:** Mary W. Donohue, willard3@umbc.edu


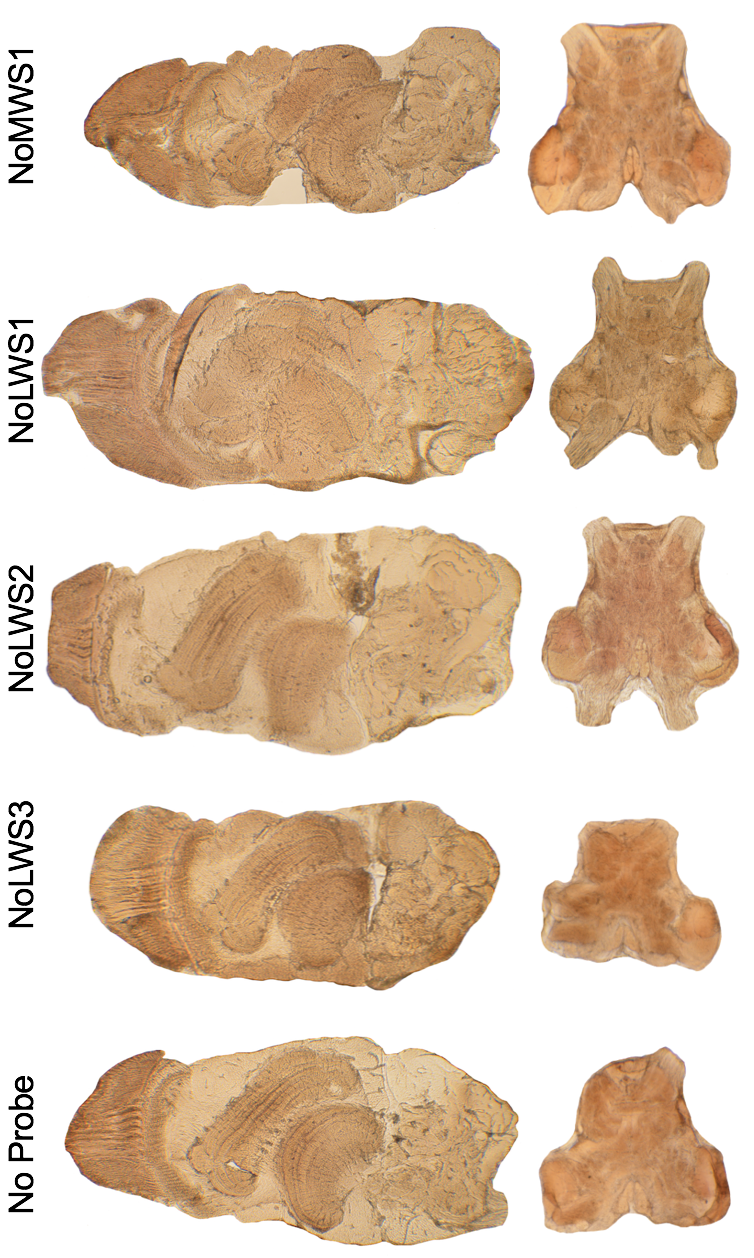


**Supplementary Figure S1.** Sense probe and no probe controls for *in situ* labeling of opsin transcripts (NoMWS1, NoLWS1, NoLWS2, and NoLWS3) in the retina, optic lobes, and cerebral ganglion of *Neogonodactylus oerstedii*.


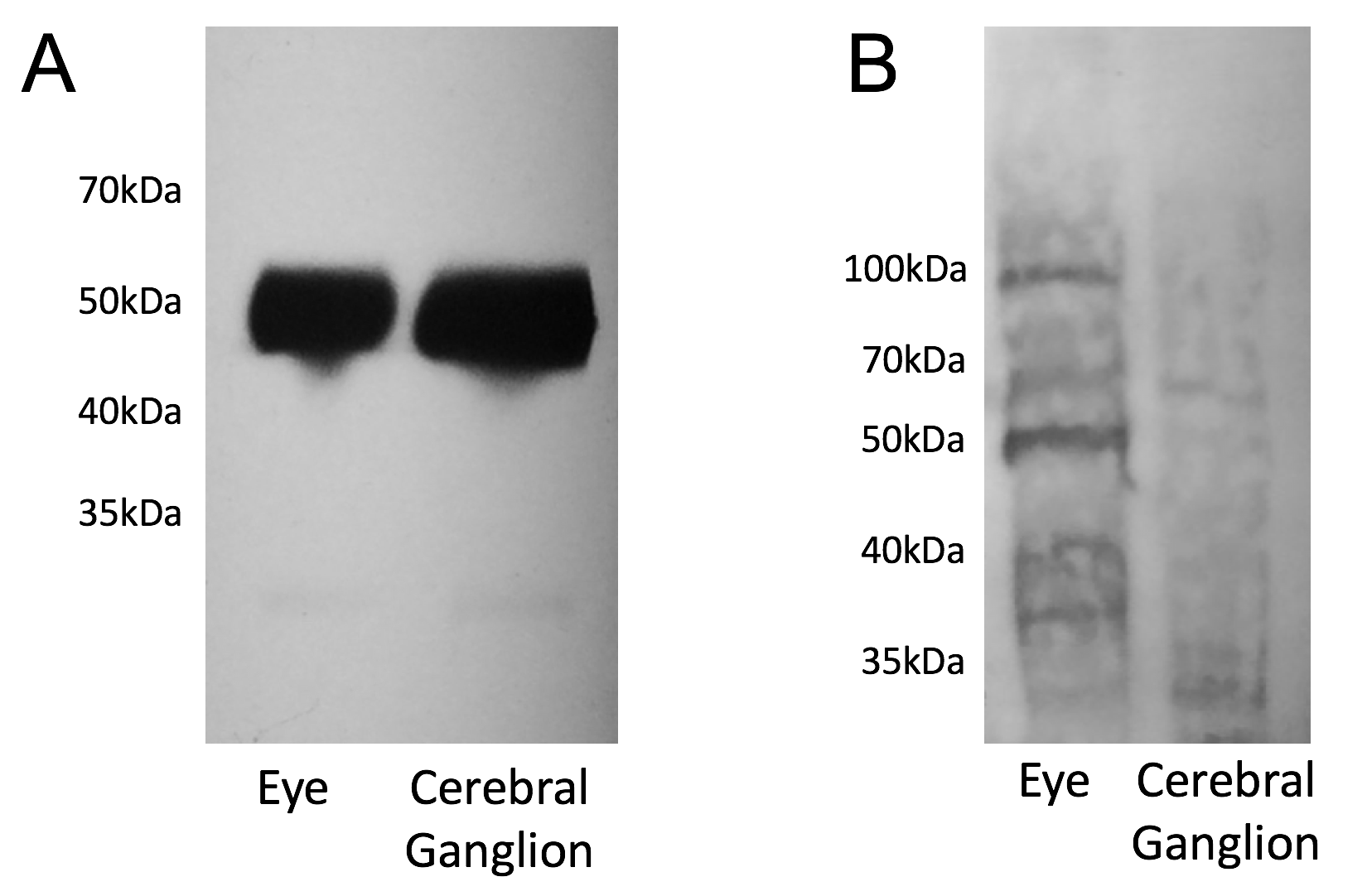


**Supplementary Figure S2.** Western blot analyses suggest that the α-tubulin antibody and crayfish long-wavelength-sensitive opsin (cLWS) antibody bind proteins of expected molecular weights that were extracted from the eye or cerebral ganglion. **A.** The α-tubulin antibody binds to protein approximately 50 kDa in weight. **B.** The cLWS antibody binds proteins that are approximately 50 kDa and 100 kDa in weight, that of other arthropod opsin monomers and dimers [22]. Bands labeling ocular proteins are much darker, probably due to the higher concentration of opsin in the retina. These black and white photographs of the western blots (run on separate gels) have not been altered to enhance contrast.

**Supplementary Figure S3.** Immunohistochemistry controls without primary antibody. **A−C.** Retinal, eyestalk, or cerebral ganglion (CG) tissues, Figure A−C respectively, were fixed in 4% paraformaldehyde overnight, and treated the same as those labeled with antibody designed against crayfish long-wavelength-sensitive (cLWS, magenta) opsin, except without incubating in primary antibody. **D−F.** Retinal, eyestalk, and CG tissues, Figures D−F respectively, were fixed in 4% carbodiimide overnight, and treated the same as those labeled with anti-histamine (white) antibody, except without incubating in primary antibody. Cell nuclei were visualized using DAPI (cyan), and all control tissue sections were imaged under 10X objective magnification by confocal microscopy.

| **Target opsin sequence** | **3′-UTR sequence used to make riboprobes** |
| --- | --- |
| **NoMWS1** | GCT GAC CTA TCT CCA CTC ACA ACT CGC TCA TGA ACT GGA ACA AGT GAC CCG AAC TTT CGC ACT TCG AAA CTC ACC C |
| **NoLWS1** | GGA AGA AAG ATG TGT CTC TGG ACC GGA AAC CGA CTA AGA ATG GAA TGA TAT TGC TAA CTG ACG ATA ACG ATA ACC GAT GAC TC |
| **NoLWS2** | GTC AGC TAC AAA TTT AAC CCC CCT TCA CAC ACA CAC ATC TCC AAG ATC C |
| **NoLWS3** | GGT AAT TCG TCT GAA GTG ATC TCC CTC TGT AAT ATA CGT CTT TTG ACC ACA TTG CAG CTC AAT GTT TGG GAG GCA ATC CAA AC |

**Supplemental Table S1.** Riboprobe sequences were designed against the untranslated region at the three prime end of the gene (3′-UTR).
